# Supplementary material for: Structural Insights for the Stronger Ability of Shrimp Ferritin to Coordinate with Heavy Metal Ions as Compared to Human H-Chain Ferritin
Source: Int J Mol Sci. 2021 Jul 23;22(15):7859. doi: 10.3390/ijms22157859 (PMC8346123; doi:10.3390/ijms22157859)
Supplement: Supplementary file 1 [file ijms-22-07859-s001.zip › ijms-1306498-supplementary.pdf]

Supplementary Materials

for

Structural insights for the stronger ability of shrimp ferritin to coordinate with heavy metal ions as compared to human H-chain ferritin

Yingjie Wang[1], Jiacheng Zang[1], Chengtao Wang[2], Xiuqing Zhang[1,], Guanghua Zhao[1,]**

[1]College of Food Science and Nutritional Engineering, China Agricultural University, Beijing Key Laboratory of Functional Food from Plant Resources, Beijing, 100083, China.

[2]Beijing Engineering and Technology Research Center of Food Additives, Beijing Technology & Business University, No. 11 Fucheng Road, Haidian District, Beijing 100048, China.

* Corresponding authors: E-mail: xiuqingzhang@cau.edu.cn; gzhao@cau.edu.cn

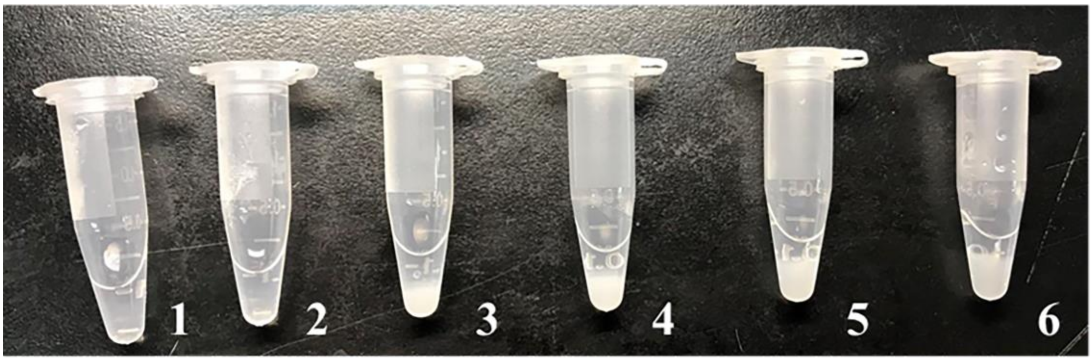

Figure S1. Solution images of HuHF plus Hg^{2+}.Tube 1: untreated HuHF sample; tubes 2, 3, 4, 5 and 6 correspond to HuHF upon treated with Hg^{2+} at a metal/protein ratio of 50/1, 100/1, 250/1, 500/1 and 1000/1, respectively.

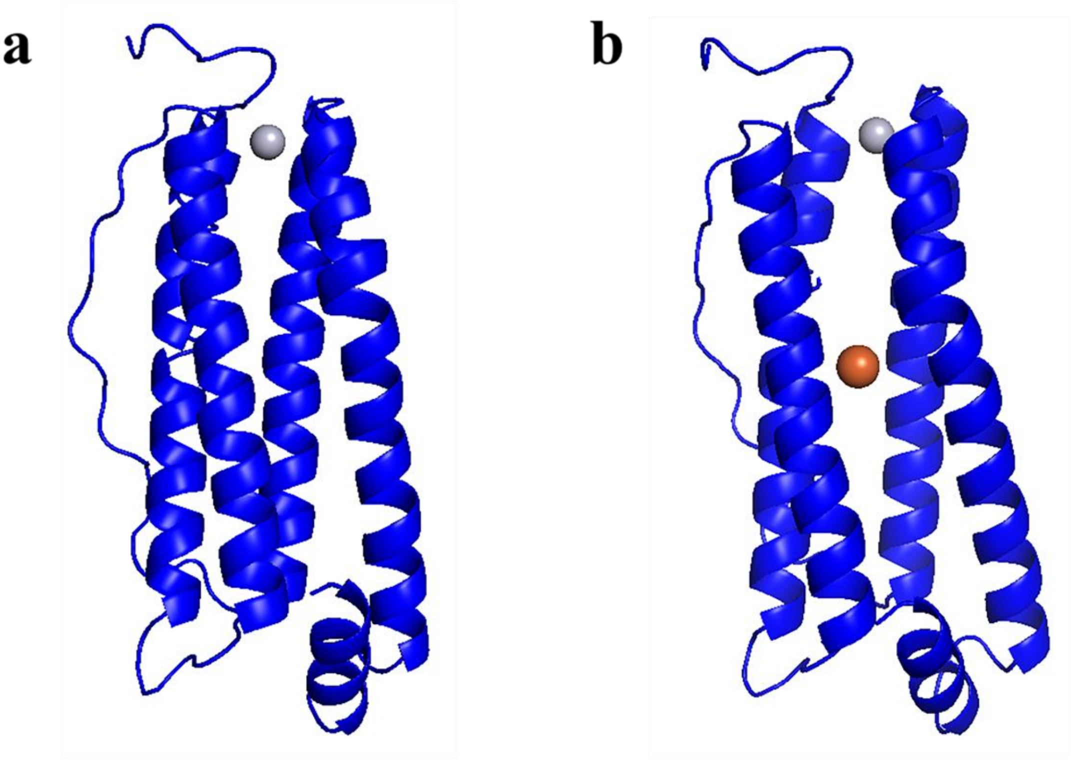

Figure S2. (a) The crystal structure of the complex of MjF and Hg^{2+} without iron near the ferroxidase site. (b) The crystal structure of the complex of MjF and Hg^{2+} with iron near the ferroxidase site. Iron is highlighted in red.

Table S1.

Parameters	MjF-Hg^{2+}	MjF-Cd^{2+}	MjF-Hg^{2+}（remove Fe）
**Data collection**			
Beamline	SSRF BL19U	SSRF BL19U	SSRF BL19U
Wavelength (Å)	0.9789	0.9789	0.9785
Space group	*I4*	*P6322*	*I4*
Unit cell	125.372, 125.372, 176.189, 90, 90, 90	155.613, 155.613, 162.729, 90, 90, 90,	126.285, 126.285, 177.247, 90, 90, 90
[a]Resolution (Å)	29.55-2.30	30.02-3.00	30.02-2.70
[a]Redundancy	3.4	10.4	3.5
[a]Completeness (%)	99.8	99.6	99.9
[a]$I/\sigma I$	5.8	8.7	6.6
[a,b]R_{pim}	0.072	0.056	0.063
[c]$CC_{1/2}$	0.982	0.990	0.976
**Refinement**			
Unique reflections	60408	23717	37908
Measured reflections	119004	43884	74922
[d]R_{work}/R_{free}	0.195 / 0.237	0.240 / 0.275	0.254 / 0.332
Wilson B-factor ($Å^2$)	29.45	43.53	47.05
B-factors ($Å^2$)	31.12	37.59	49.44
Rms. deviations			
Bond lengths (Å)	0.001	0.009	0.011
Bond angles (°)	1.01	1.145	1.311
Ramachandran plot (%)			
Favored	98.38	97.46	92.41
Outliers	0.10	0.15	1.83

[a]Highest resolution shell is shown in parentheses.

[b]$R_{pim}=\sum_{hkl} [1/(N-1)]^{1/2}\sum_j |I_j(hkl)-\langle I(hkl)\rangle| / \sum_{hkl} \sum_j I_j(hkl)$, where N is the redundancy of the dataset.

[c]$CC_{1/2}$ is the correlation coefficient of the half datasets.

[d]$R_{work} = \sum_{hkl} | |F_{obs}| - |F_{calc}| | / \sum_{hkl} |F_{obs}|$, where F_{obs} and F_{calc} is the observed and the calculated structure factor, respectively. R_{free} is the cross-validation R factor for the test set of reflections (5% of the total) omitted in model refinement.
